# Secondary congenital aphakia

**Published:** 2016

**Authors:** Catalina Ionescu, Dana Dascalescu, Miruna Cristea, Speranta Schmitzer, Miruna Cioboata, Raluca Iancu, Catalina Corbu

**Affiliations:** * Clinical Ophthalmology Emergency Hospital, Bucharest, Romania; ** University Emergency Hospital Bucharest, Bucharest, Romania

**Keywords:** congenital cataract, rubella, congenital aphakia

## Abstract

Purpose: We present the clinical, paraclinical and therapeutic features in a patient with secondary congenital aphakia.

Methods: A 2-year-old patient, diagnosed with congenital rubella syndrome including sensorineural deafness, congenital heart disease, intellectual disability, microcephaly, microphthalmia, and congenital cataract, presented to our clinic for the surgical treatment of cataract.

Results: During the surgery, the absence of the lens’ cortex was observed, hence, the final diagnose was of secondary congenital aphakia. Surgery was then continued with a posterior capsulorhexis and an anterior vitrectomy, deciding to postpone the implantation of the posterior chamber intraocular lens.

## Introduction

Aphakia is defined as the absence of the lens. Aphakia can be congenital due to the intrauterine anomalies and acquired due to the surgical removal or trauma. Congenital aphakia is a rare anomaly, which can be associated with other important ocular disorders. It can be subdivided into two forms: primary and secondary congenital aphakia. Primary congenital aphakia results from the failed induction of the lens placode and therefore the lens is absent, whereas in secondary aphakia, the lens placode has developed but has been resorbed before birth (remnants of the lens such as the lens capsule are present) [**1**,**2**].

The lens development begins in the 3rd- 4th week of gestation, when the thickening of the surface epithelial cells over the optic vesicle gives birth to the optic placode, which, after the process of invagination, becomes the lens vesicle. The latter contains a single layer of cells covered by a basal lamina, which will ultimately form the lens capsule. In the 3rd month, the primary lens fibers begin to fill the cavity from posterior to anterior [**1**].

**Table 1:** Etiology [**3**]

Congenital cataract	Congenital aphakia
Genetically transmitted syndromes and metabolic disorders (Down Sd., Marfan Sd., Lowe Sd., galactosemia)	Genetically transmitted disorders (FOXE3 gene mutation, PAX6 gene mutation)
Infectious causes (rubella, toxoplasmosis, CMV, HIV)	Infectious causes (rubella)
Other:hypoglycemia, corticosteroids administration during pregnancy	Less known

## Methods

A 2-year-old patient with a medical history of congenital rubella syndrome, which included bilateral congenital cataract, operated congenital heart disease (ventricular and atrial septal defects, pulmonary artery stenosis), mental retardation, hypotonia, sensorineural hearing loss with cochlear implant and dental abnormalities was admitted to our clinic for reevaluation and surgical treatment of congenital cataract.

The clinical examination was difficult to be performed due to the mental retardation. External and slit-lamp examination of the anterior segment revealed esotropia, horizontal pendulum oscillations in both eyes, microcornea and nonhomogeneous anterior capsular opacities (RE>LE). Fundus examination could not be performed.

The B-Scan ultrasonography showed an axial length of 16,5 mm (right eye) and 17 mm (left eye), attached retina. It also revealed reflective membranous echoes with a narrow space that suggested the presence of the lens capsule. The corneal diameter measured 8 mm.

**Fig. 1:**
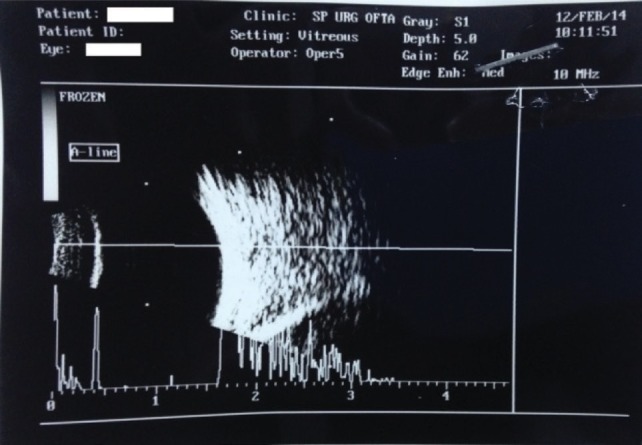
Right eye: reflective membranous echoes

**Fig. 2:**
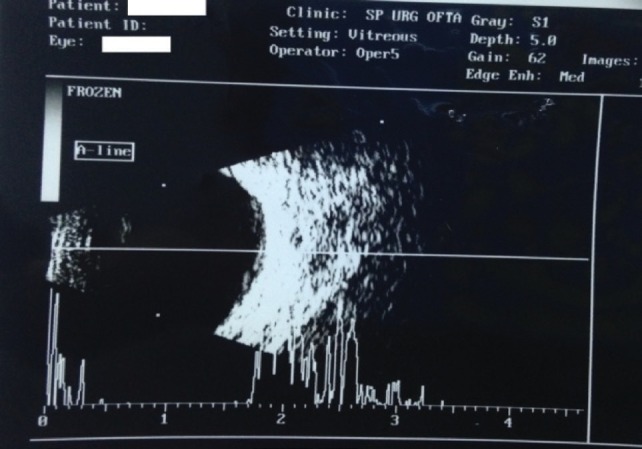
Left eye: membranous echoes (the possibility of an incomplete posterior capsule)

The preliminary diagnosis at that moment was congenital cataract, microphthalmia, esotropia, and horizontal nystagmus in both eyes. It was decided that cataract surgery should be performed for the right eye and the implantation of the posterior chamber intraocular lens should be delayed because the patient presented microphthalmia.

Because of a small pupil, iris retractors had to be set up intraoperatively. Next, the anterior capsulorhexis was challenging due to a fibrotic capsule. Surprisingly, after that step, the absence of the lens’ cortex was ascertained and the surgery was continued with a posterior capsulorhexis and an anterior vitrectomy. The surgery had no postoperative complications and it was decided that the other eye should be operated after one month, but the patient was admitted one year later for an intervention at the left eye, which had a similar course as the one in the right eye. During the surgery, the same congenital anomaly was observed, hence the final diagnosis was of secondary congenital aphakia of infectious etiology based on serologic tests (mother positive for rubella virus, rubella-specific IgM blood test positive in the child), intraoperative findings and ophthalmic ultrasound.

## Discussion

In these cases, surgery does not solve everything. The next step and probably the most important and difficult to be achieved is the visual rehabilitation, which can be obtained first with posterior chamber intraocular lens’ implantation, followed by contact lenses and optical correction with spectacles. In our case, we thought that the best solution was the optical correction with spectacles (+15 Dsf) and ocular occlusion as therapy for amblyopia.

**Fig. 3:**
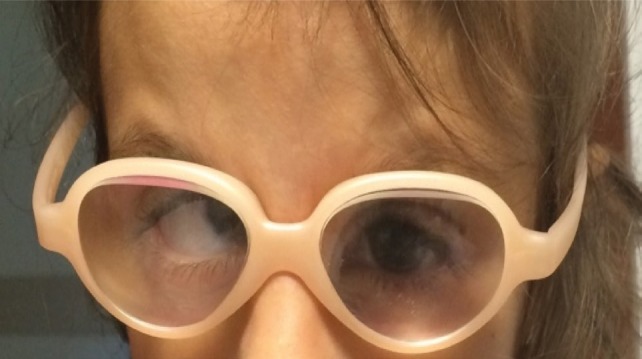
Patient after cataract surgery with the given optical correction

The other two options were excluded because it was impossible to implant a posterior chamber intraocular lens, due to the patient’s microphthalmia and the contact lenses were too large for the patient’s microcornea.

The long period between the two surgeries was responsible for amblyopia and the worsening of the existent esotropia. Also, the patient was noncompliant due to mental retardation and, in addition, the cochlear implant represented a mechanical obstacle in wearing the spectacles.

The possibility of a future implantation of a posterior chamber lens if the eye reached an appropriate axial length was taken into account.

Although the rubella virus generally has a benign evolution in children and adults, it has important vital consequences if contracted cardiac defects, deafness, and mental retardation, especially if the infection occurs during the first trimester of pregnancy [**4**]. The rubella virus has an apoptotic effect on the primary lens fibers during pregnancy but it may persist in the lens three years postnatally [**5**].

## Conclusions

Congenital cataract surgery is a challenge for every surgeon because of the different anatomic variations of the patients and the poor visual outcome even after the surgical treatment. In our case, the presumed congenital cataract was in fact a secondary congenital aphakia associated with a multitude of other systemic malformations caused by rubella virus.

The occlusion therapy of the patient’s amblyopia is the key for a good visual result in the future. Also, since the patient has a considerable risk of developing secondary glaucoma, the screening of glaucoma becomes an important step in evaluating the long-term outcome of the patient [**6**].
